# Turbulent flow as a cause for underestimating coronary flow reserve measured by Doppler guide wire

**DOI:** 10.1186/1476-7120-4-14

**Published:** 2006-03-22

**Authors:** Markus Ferrari, Gerald S Werner, Philipp Bahrmann, Barbara M Richartz, Hans R Figulla

**Affiliations:** 1 Clinic of Internal Medicine I, Friedrich-Schiller-University, Jena, Germany; 2 Clinic of Internal Medicine, City Clinical Center Darmstadt, Germany

## Abstract

**Background:**

Doppler-tipped coronary guide-wires (FW) are well-established tools in interventional cardiology to quantitatively analyze coronary blood flow. Doppler wires are used to measure the coronary flow velocity reserve (CFVR). The CFVR remains reduced in some patients despite anatomically successful coronary angioplasty. It was the aim of our study to test the influence of changes in flow profile on the validity of intra-coronary Doppler flow velocity measurements in vitro. It is still unclear whether turbulent flow in coronary arteries is of importance for physiologic studies in vivo.

**Methods:**

We perfused glass pipes of defined inner diameters (1.5 – 5.5 mm) with heparinized blood in a pulsatile flow model. Laminar and turbulent flow profiles were achieved by varying the flow velocity. The average peak velocity (APV) was recorded using 0.014 inch FW. Flow velocity measurements were also performed in 75 patients during coronary angiography. Coronary hyperemia was induced by intra-coronary injection of adenosine. The APV maximum was taken for further analysis. The mean luminal diameter of the coronary artery at the region of flow velocity measurement was calculated by quantitative angiography in two orthogonal planes.

**Results:**

In vitro, the measured APV multiplied with the luminal area revealed a significant correlation to the given perfusion volumes in all diameters under laminar flow conditions (r^2 ^> 0.85). Above a critical Reynolds number of 500 – indicating turbulent flow – the volume calculation derived by FW velocity measurement underestimated the actual rate of perfusion by up to 22.5 % (13 ± 4.6 %). In vivo, the hyperemic APV was measured irrespectively of the inherent deviation towards lower velocities. In 15 of 75 patients (20%) the maximum APV exceeded the velocity of the critical Reynolds number determined by the in vitro experiments.

**Conclusion:**

Doppler guide wires are a valid tool for exact measurement of coronary flow velocity below a critical Reynolds number of 500. Reaching a coronary flow velocity above the velocity of the critical Reynolds number may result in an underestimation of the CFVR caused by turbulent flow. This underestimation of the flow velocity may reach up to 22.5 % compared to the actual volumetric flow. Cardiologists should consider this phenomena in at least 20 % of patients when measuring CFVR for clinical decision making.

## Background

Cardiologists are able to quantify the coronary flow velocity during coronary angiography by means of a Doppler guide wire system. The computer analysis of the Doppler shift reflects the actual blood flow velocity in the coronary artery [1]. In addition, measuring the coronary flow velocity reserve (CFVR) during pharmacological stimulation of coronary blood flow can characterize the vaso-dilatory capacity of the coronary arteries [2]. The CFVR is the ratio of hyperemic flow velocity divided by baseline flow velocity. It correlates with the functional severity of epicardial coronary artery stenosis [3,4]. The CFVR is well established for physiologically guided decision making in the catheter laboratory [5-7]. The results of interventional and of medical treatment in coronary artery disease have been assessed by intra-coronary Doppler studies [8,9]. An impaired CFVR was described after angiographically successful coronary angioplasty despite lack of residual stenosis [10]. Various patient-related factors such as coronary micro-embolization, coronary spasm, micro-thrombi or endothelial dysfunction have been discussed as possible causes for an impaired flow velocity reserve measured by Doppler wire [11,12]. Assuming a laminar flow profile, volumetric flow analysis by means of a Doppler-tipped wire showed a high correlation between the real flow and the measured Doppler-shift in vitro and in vivo [13,14]. However, direct analysis of coronary flow pattern in open heart surgery showed the occurrence of turbulent flow even in non-atherosclerotic coronary arteries [15]. Axial-directed beam studies with Doppler probes revealed a limitation of the accuracy of Doppler flow velocity measurements related to the occurrence of turbulent flow [16,17]. The critical Reynolds number (CRN) indicates the flow velocity, which discriminates between laminar and turbulent flow according to vessel diameter. Above a CRN of 80 – 710 the flow profile of circulating blood changes from laminar to turbulent [18]. We hypothesized that turbulent flow may also occur in human coronary arteries influencing Doppler flow measurements. To prove this hypothesis, we performed in vitro experiments to examine the influence of the flow profile on the shift of the Doppler signal. The occurrence rate of high flow velocities above the CRN assuming the presence of turbulent flow was investigated by in vivo studies.

## Methods

We used 0.014 inch Doppler-tipped guide wires (FloWire™, Cardiometrics, Carolina, USA) for the in vitro, and for the in vivo study. A piezo-electric ultrasound transducer was integrated in the tip of the 175 cm long wire. The signal of the 12 MHz pulsed Doppler was analyzed by a computer system (FlowMap™, Cardiometrics) using fast Fourier transformation. Technical details of the Doppler System were described elsewhere [19].

### In vitro experiments

We used glass pipes of a length of 20 cm in the in vitro model. They had defined inner diameters of 1.5, 2.5, 3.5, 4.5, and 5.5 mm to simulate coronary vessel. The glass pipes were perfused by a roller pump with heparinized blood from volunteer donors. Careful attention was paid for an exact calibration of the pump flow before each measurement using volumetric calibration. Setting different pump speeds according to physiologic velocities of coronary blood flow, the mean flow velocity was varied between 5 and 180 cm/s. The motor currency of the roller pump was directly supervised by a personal computer via an analog-digital interface. Careful attention was paid to keep the motor currency stable during all flow velocity recordings. Twenty measurements were taken at each flow velocity after repositioning of the Doppler wire in the mid segment of the glass pipes. The wire was twisted and moved until a stable signal was obtained before each measurement. The mean value of the average peak velocity (APV) sampled by continuous recording over a two minute period was taken for further analysis at each perfusion speed.

**Figure 1:**
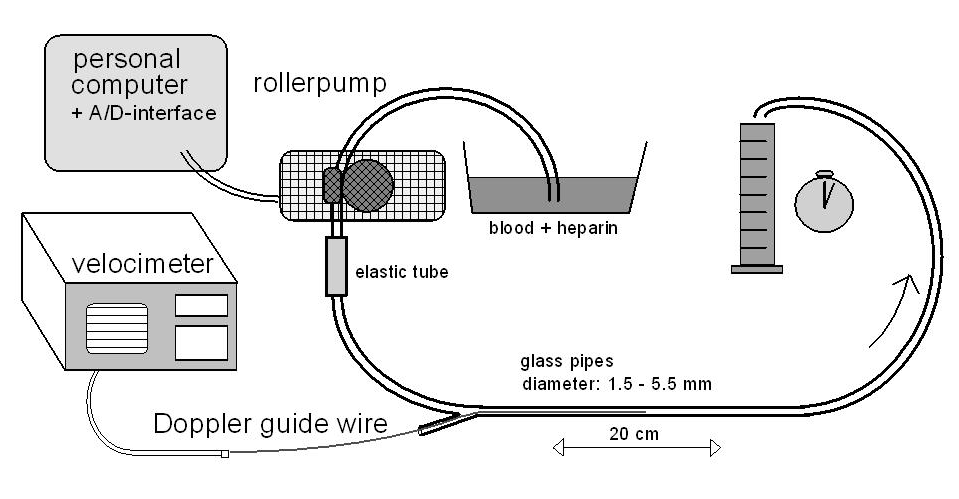
Setup for the in vitro measurements. Heparinized blood was pumped through glass pipes of diameters of 1.5 to 5.5 mm at defined flow rates.

**Figure 2:**
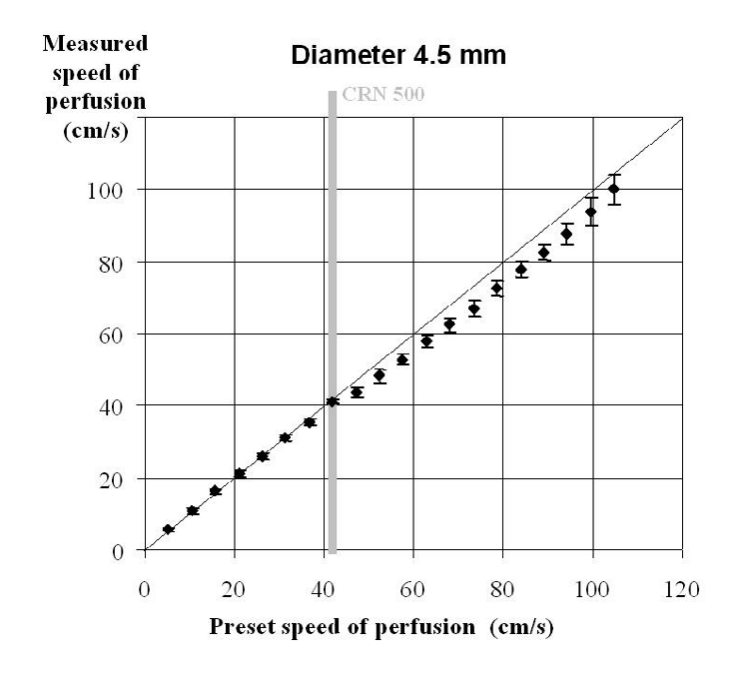
Mean values and standard deviations of 20 in vitro Doppler flow measurements at each per perfusion speed in glass pipes of 4.5 mm diameter. The flow velocity of the critical Reynolds number (CRN) 500 is marked.

**Figure 3:**
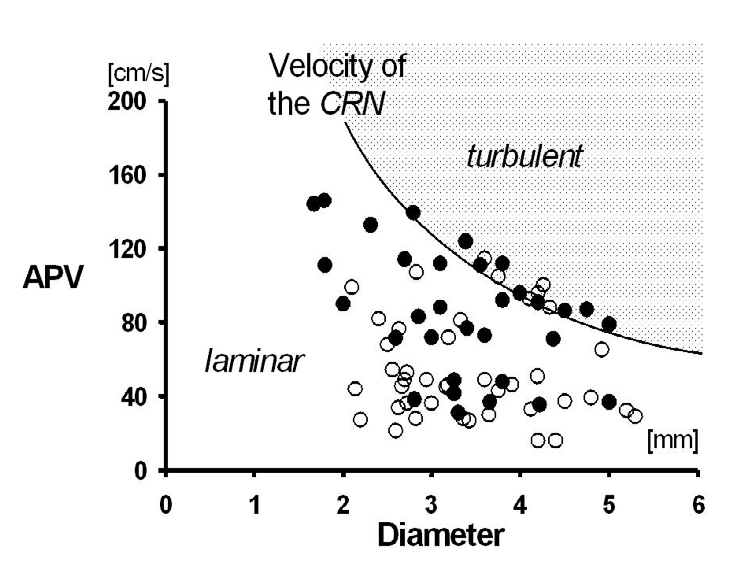
Maximum value of the average peak velocity APV in 43 patients before (○) or after an angiographically successful angioplasty of 32 patients (●). The highest values of APV were taken for each individual. The flow velocity at the critical Reynolds number (CRN) 500 describes the change from laminar to turbulent flow profile.

**Figure 4:**
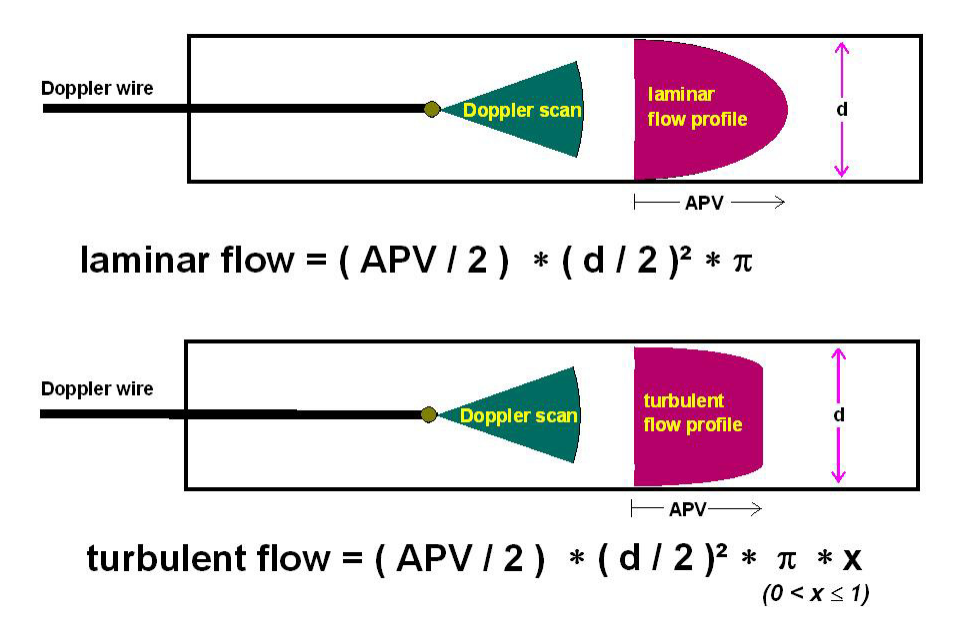
Flow profile under laminar and turbulent conditions: Under laminar flow the average peak velocity divided by 2 (APV/2) multiplied with the lamina area (vessel diameter = d) can be used for exact quantitative measurement of the blood flow. Under turbulent flow an underestimation of the blood flow occurs when measured with a Doppler wire.

### Measurements of coronary flow velocity in vivo

#### Patient selection

All patients participating in this study, were scheduled for an elective coronary angiography due to pathological findings in non-invasive stress test, and stable angina pectoris. They had given informed consent for coronary angiography, and for percutaneous coronary intervention (PCI) if necessary. All lesions were de novo lesions. None of the patients had undergone previous PCI of the target vessel. To have a similar blood viscosity in all patients, careful attention was paid on a normal (within the normal range) hematocrit value in all study patients before coronary angiography. All patients had also given written informed consent to participate in the study. They were well informed about intra-coronary flow velocity measurements. The study protocol was approved by the ethical committee of our University. Aortic stenosis, arterial hypertension (2^nd ^degree or above), and other co-morbidities influencing the blood viscosity (e.g. leukemia) were exclusion criteria.

#### Angiography protocol

Coronary angiography was performed in Judkin's technique by a femoral approach through a 7F sheath. A standard guiding catheter was placed with its tip in the coronary ostium of the target vessel. The 0.014" Doppler guide wire was placed 1 – 2 cm distal to the stenosis after intravenous administration of 10.000 IU of heparin.

After intra-coronary injection of 0.2 mg nitroglycerin quantitative coronary angiography (QCA) of the target lesion was obtained. The QCA was performed in two orthogonal projections by Philips DCI automated coronary analysis using the 7F guiding catheter as reference standard [20]. Lesion severity was determined as percentage diameter stenosis relative to the angiographically normal diameter. Mean values of the vessel diameter 3 to 5 mm distal to the tip of the Doppler wire of the two planes were taken for further analysis.

An adenosine bolus was injected through the guiding catheter to induce maximum hyperemic blood flow after registration of the APV at rest. We used 12 μg adenosine for the right, and 18 μg adenosine for the left coronary artery. The CFVR was computed as the ratio of hyperemic to basal average peak velocity (APV). A PCI was performed in those patients with an impaired CFVR below 2.0. Balloon inflations were repeated or a stent was implanted until an angiographically satisfying result was achieved in all patients. The maximum values of the APV were taken from each patient for further analysis.

### Calculations and statistical analysis

The Reynolds number (Re) can be calculated from the mean luminal diameter (MLD) multiplied with the flow velocity (APV/2) and the density (Q) divided by the viscosity of blood (n):



MLD = mean luminal diameter, APV/2 = mean of average peak velocity, Q = density of blood (1,060 kg*m^-3^), n = viscosity of blood (4*10^-3 ^Pa*s).

The laminar flow profile of blood changes to a turbulent flow above the CRN. The flow velocity of the CRN was calculated using the MLD which was obtained by QCA in all patients. Blood flow rate (BF) was calculated from the APV divided by 2 and the luminal area (LA) of the vessel (π*(MLD/2)^2^) [21]:

BF = LA * APV/2 = APV/2* π*(MLD/2)^2^

BF = blood flow, LA = luminal area (π* vessel radius ^2^); APV/2 = mean of average peak velocity, MLD = mean luminal diameter.

The correlation coefficient (r) and its square value (r^2^) were obtained by computerized calculation with Excel 2000 (Microsoft Corp.). All values are expressed as mean ± standard deviation.

## Results

### In vitro studies

The BF measured by Doppler showed excellent correlations to the given perfusion speeds in all diameters under laminar flow conditions: we calculated an r^2 ^– value of 0.854 in the 1.5 mm system, in the 2.5 mm glass pipes r^2 ^was 0.957, in the 3.5 mm diameter we found an r^2 ^of 0.968, in the 4.5 mm system it was 0.989, and in the 5.5 mm tubes we obtained an r^2 ^– value of 0.991 respectively. The difference between the actual volume flow and the flow volume calculated from the Doppler signal was below ± 4.2 % in all measurements under laminar perfusion (Table 1). According to our measurements, we calculated a critical Reynolds (CRN) number of about 500 (493 – 509). At flow velocities above the CRN (= turbulent flow) the velocity measured by the Doppler wire was up to 12.8 % lower in 1.5 mm glass pipes, up to 22.5 % lower in 2.5 mm glass pipes, up to 14.7 % lower in 3.5 mm glass pipes, up to 8.9 % lower in 4.5 mm glass pipes, and up to 13.1% lower in 5.5 mm glass pipes compared to the quantitatively measured perfusion volumes. The average underestimation of blood flow under turbulent conditions was 13 ± 4.6 % varying between 1.8 % and 22.5 %.

**Table 1:** Mean values ± standard deviation of 20 in vitro Doppler flow measurements of the average peak velocity at each perfusion speed in glass pipes of 1.5 – 5.5 mm. The perfusion speed was adjusted up to 700 ml/min (2.5 – 5.5 mm diameter) and up to 200 ml/min in pipes of 1.5 mm diameter. The flow velocity was calculated of the blood flow (Perf.) and the luminal area of the pipe. The velocity value at the critical Reynolds number () is marked for each diameter.

*Diameter:*	*1.5 mm*			*2.5 mm*			*3.5 mm*			*4.5 mm*			*5.5 mm*		
Velocity of CRN	125.8 cm/s			75.5 cm/s			53.9 cm/s			41.9 cm/s			34.3 cm/s		

Perf. (ml/min)	Velocity (cm/s)	APV		Velocity (cm/s)	APV		Velocity (cm/s)	APV		Velocity (cm/s)	APV		Velocity (cm/s)	APV	
		Mean	± SD		Mean	± SD		Mean	± SD		Mean	± SD		Mean	± SD

50	47.2	94.7	± 1.23	17.0	35.7	± 0.65	8.7	17.4	± 0.48	5.2	11.4	± 0.48	3.5	7.1	± 0.08
100	94.3	183.7	± 1.56	34.0	68.0	± 0.63	17.3	34.1	± 0.97	10.5	21.6	± 0.73	7.0	14.0	± 0.0
													
150	141.5	259.6	± 3.88	50.9	97.0	± 1.52	26.0	51.6	± 0.73	15.7	32.6	± 0.66	10.5	20.7	± 0.57
200	188.6	328.9	± 7.56	67.9	120.5	± 2.38	34.7	67.1	± 0.67	21.0	42.3	± 0.71	14.0	27.2	± 0.51
													
250				84.9	145.2	± 3.79	43.3	82.1	± 0.77	26.2	51.7	± 0.78	17.5	33.5	± 0.87
300				101.9	169.6	± 5.28	52.0	96.5	± 0.97	31.4	62.3	± 0.78	21.1	39.1	± 1.56
													
350				118.8	195.3	± 6.91	60.6	110.5	± 0.80	36.7	70.9	± 0.73	24.6	44.5	± 2.36
													
400				135.8	220.8	± 8.29	69.3	124.3	± 1.61	41.9	82.3	± 0.71	28.1	50.8	± 2.58
450				152.8	246.7	± 11.04	78.0	140.7	± 0.90	47.2	87.1	± 1.34	31.6	55.6	± 3.41
													
500				169.8	271.1	± 12.50	86.6	152.1	± 1.51	52.4	96.4	± 1.96	35.1	61.9	± 2.74
550				186.7	296.5	± 14.73	95.3	163.6	± 1.62	57.6	105.5	± 1.57	38.6	67.1	± 3.63
600				203.7	340.6	± 14.79	103.9	178.8	± 2.32	62.9	115.6	± 1.83	42.1	73.4	± 3.98
650				220.7	362.6	± 15.40	112.6	192.7	± 1.56	68.1	124.8	± 1.96	45.6	79.5	± 4.10
700				237.7	368.3	± 25.65	121.3	206.9	± 2.08	73.4	133.8	± 2.32	49.1	85.8	± 4.91

### In vivo measurements

It was the aim of our study to transfer the in vitro findings to the clinical routine of intra-coronary flow velocity measurements in diseased coronary arteries of patients in the catheter laboratory. It was unclear whether these critical flow velocities could occur in a relevant number of patients. However, these measurements were performed irrespectively of the inherent deviation caused by the described phenomena.

A total of 75 patients (58 men = 77.3 %) were studied with an average age of 59.5 ± 11.2 years. The baseline characteristics, and the results of the flow velocity measurements of the study patients are summarized in Table 2.

**Table 2:** Data of 75 patients undergoing elective coronary angiography: The degree of stenosis was calculated by quantitative angiography in two orthogonal planes. Mean values ± standard deviations (SD), APV: highest value of the average peak velocity (under baseline conditions and maximal hyperemia), the coronary flow velocity reserve was measured before angioplasty (CFVR before PCI) in 75 patients, and after angioplasty (CFVR post PCI) in 42 patients.

		**Mean**	± **SD**
**Age**	(years)	59.5	± 11.2
**Degree of stenosis**	(%)	61.7	± 9.2
**APV_base_**	(cm/s)	34.2	± 17.87
**APV_max_**	(cm/s)	67.3	± 33.65
**CFVR_before PCI_**	(n = 75)	2.0	± 0.71
**CFVR_post PCI_**	(n = 42)	2.7	± 0.35

The flow velocity was measured in 23 patients (31 %) in the right coronary artery (RCA), in 18 patients (35 %) in the circumflex artery, and in 34 patients (65 %) in the left anterior descending artery. A PCI was necessary in 42 patients (56 %). In these patients the CFVR increased from 1.6 ± 0.40 to 2.7 ± 0.35 after PCI. Only 12 patients received a stent due to an angiographically unsatisfying result of the balloon inflation. We measured the maximum hyperemic APV value in 43 patients before, and in 32 patients after an angioplasty. Among those 43 patients were 33 patients (77 %) in whom the angioplasty was deferred due to an initially recorded CFVR above 2.0. Peak APV values were measured in 32 patients (76 %) post angioplasty. In 10 patients (24%) the recording of the APV reached its maximum before the intervention. However, the highest individual APV value of each patient was used for further analysis. Figure 3 summarizes the maximum values of the hyperemic APV of all patients.

Peak values of APV varied between 14 cm/s and 154 cm/s. In 15 patients (20 %) the maximum APV exceeded the velocity of the CRN. In 7 patients (9.3 %) the maximum APV in the RCA, and in 8 patients (10.7 %) the maximum APV in the left coronary artery exceeded the velocity of the CRN. The average maximum values of the APV were 79 ± 17.2 cm/s after PCI (n = 32), and 61 ± 14.7 cm/s before angioplasty (n = 43).

## Discussion

We proved the hypothesis that the occurrence of turbulent flow influences the accuracy of Doppler flow velocity measurements by a combined in vitro and in vivo study. About 20% of patients have coronary flow velocities above the critical Reynolds number during pharmacologically induced hyperemia. We therefore assume that a turbulent flow profile can occur under in vivo conditions resulting in an underestimation of the coronary blood flow.

### In vitro measurements

We were able to demonstrate an excellent correlation between perfusion volume and the calculated volume derived from Doppler flow velocity measurement under in vitro conditions at low flow velocities as previously described by other research groups [22]. At flow velocities above the CRN of about 500, flow volume calculations based on the shift of the Doppler signal showed a tendency to underestimate the blood flow as illustrated by Figure 4. This phenomena can be explained by our hypothesis that the Doppler wire measured the true peak value of APV under turbulent conditions, since the intra-luminal peak APV is diminished under turbulent flow conditions. It does not reach the same value which would have been recorded with equal perfusion volumes under laminar flow conditions.

The amount of the mean underestimation of the flow velocity measured by Doppler wire was 13.1 % under relatively high perfusion speeds above the CRN. The inaccuracy of the Doppler wire was 4.2% under laminar flow. These results qualify the Doppler wire as a valid, and highly exact tool for quantitative coronary blood flow measurements under low flow velocities associated with a laminar flow profile. The CRN is reciprocally dependent on the vessel diameter given by the MLD. The velocity at the value of the CRN exceeds the normal physiological range in vessels with an MLD below 2 mm. Thus, we would suggest to place the Doppler wire in a distal non-branching segment of the coronary artery to achieve the highest accuracy of the flow velocity measurement. On the other side, the accuracy of measuring the MLD by QCA is limited in smaller and more calcified vessels [23].

### In vivo measurements

According to an inaccuracy of only ± 4.2 % under laminar flow conditions we regard intra-coronary Doppler wires as a valid tool for quantification of coronary blood flow at rest. However, several studies investigated hyperemic blood flow after pharmacological stimulation by papaverine or adenosine. Under resting conditions, we did not observe flow velocities which were above the velocity at values of the CRN. But under hyperemic conditions, about 20 % of patients reached flow velocities above the velocity of the CRN after injection of adenosine. Based on our in vitro observations, we assume that the Doppler shift derived flow velocity underestimates the coronary peak blood flow in one fifth of patients by about 13 %.

Measurements of CFVR can be relevantly influenced by a maximum APV above the CRN. For example, the measured CFVR would be 2.3 instead of 2.6 due to the occurrence of turbulent flow during hyperemia after an angiographically successful PCI. According to the results of the DEBATE II Study, the responsible cardiologist would expect a higher restenosis rate in this patient, considering the implantation of a stent [24]. The APV values at rest were below the velocity of the CRN in all patients. Thus, an overestimation of the CFVR related to an underestimated baseline APV does not appear very likely to happen under regular flow conditions.

Novel technologies like the so-called fractional flow reserve (FFR), or intra-coronary thermo-dilution can quantify high coronary blood flow with predominantly turbulent character independently of the present flow profile [25,26]. One may speculate, that changes of coronary flow profile from laminar to turbulent might have been one reason for previously reported discordance between CFVR and FFR [27]. Since turbulent flow occurs when reaching relatively high flow velocities, and healthy subjects without coronary artery disease show higher flow velocities in pharmacological tests compared to patients suffering from coronary artery disease, we assume that turbulent flow may be observed more often in healthy vessels [28]. However, measuring CFVR in diseased vessels results in relatively low peak flow velocities, therefore the chance of turbulent flow interfering with the Doppler measurements may be of less importance.

The in vitro evaluation of the CRN was performed using smooth glass pipes. In contrast to the ideal situation inside glass pipes we assume an even lower CRN in vivo when measuring APV in calcified coronary vessels. Therefore, an underestimation of flow velocity recordings might happen more often in patients than suggested by our in vitro experiments. However, we do not expect a CRN much below 500 which could also influence the accuracy of intra-coronary Doppler flow measurements at rest [29].

The velocity of the CRN depends on the vessel diameter. Saphenous vein bypass grafts show higher diameters compared to native coronary arteries which can reach MLD values of up to 8 mm. Relevant variations of the velocity distribution in vein bypass graft were uncovered by laser Doppler anemometry [30]. Thus, the flow velocity of the CRN may be reached more often in vein grafts than in native coronary vessels. Flow velocity measurements in a bifurcation of a coronary artery may also influence the quality of the flow profile [31].

Recently, coronary flow reserve was also analyzed in transthoracic Doppler studies [32]. Further studies should evaluate the influence of turbulent flow on flow velocity measurements with transthoracic Doppler.

### Limitations of the study

We were able to demonstrate an effect of high flow velocities on the validity of blood flow measurements with Doppler wires in smooth glass pipes. In contrast to coronary arteries these pipes did not have any elastic component, nor bending, calcification of the wall, local dissections, previously implanted stents, intramural thrombus, side branches, or changes in vessel diameter which all have an influence on the flow profile possibly causing turbulent flow [33,34].

Especially the elastic component, changes in diameter, and the hematocrit influencing the viscosity of the blood might have a major influence on the CRN in human coronary arteries [35,36]. The derived blood flow was underestimated only at high flow velocities under in vitro conditions. In a study population of 75 patients, 20 % achieved such high flow velocities above the CRN during maximal hyperemia. Today, higher doses of adenosine are given i. c. for induction of hyperemia [37]. Therefore, the proportion of patients achieving coronary peak flow velocities above the value at the CRN may be even higher due to this more intensive pharmacological stimulation. As mentioned above, in comparison to smooth and straight glass pipes, turbulent flow might occur more often in coronary arteries which show an increasing proportion of previously implanted stents. On the other hand, the elastic vessel wall can reduce the amount of turbulent flow. However, we see limitation of transferring the results of the in vitro measurements directly to the in vivo Doppler studies.

## Conclusion

Below the critical Reynolds number Doppler guide wires are a highly exact and valid tool for quantitative measurements of coronary blood flow velocity. If the flow velocity exceeds the critical Reynolds number of 500, Doppler flow velocity measurements underestimate the actual flow by 13 %. An overestimation of the flow does not appear possible based on this phenomena. Turbulent flow occurs after pharmacological stimulation of the coronary flow blood in at least 20 % of patients which could lead to an underestimation of the Doppler velocity shift derived coronary flow velocity reserve. This phenomena of turbulent flow influencing the accuracy of flow velocity measurements should therefore be taken into consideration in all studies analyzing Doppler derived coronary blood flow during hyperemia.

## Competing interests

The author(s) declare that they have no competing interests.

## Authors' contributions

MF, PB, and BMR performed the in vitro studies. MF, GSW, and HRF performed the in vivo studies. All authors provided major scientific input into the study, and reviewed the manuscript. HRF was the scientific supervisor of the project. MF, GSW, and BMR were responsible for the design of the study, and carried out all statistical analyses. MF is the corresponding author. All authors read and approved the final version of the manuscript.
